# The Genome of *Staphylococcus epidermidis* O47

**DOI:** 10.3389/fmicb.2020.02061

**Published:** 2020-08-25

**Authors:** Stefan Raue, Sook-Ha Fan, Ralf Rosenstein, Susanne Zabel, Arif Luqman, Kay Nieselt, Friedrich Götz

**Affiliations:** ^1^Institute for Bioinformatics and Medical Informatics, University of Tübingen, Tübingen, Germany; ^2^Microbial Genetics, Interfaculty Institute of Microbiology and Infection Medicine Tübingen (IMIT), University of Tübingen, Tübingen, Germany; ^3^Infection Biology, Interfaculty Institute for Microbiology and Infection Medicine Tübingen (IMIT), University of Tübingen, Tübingen, Germany; ^4^Biology Department, Institut Teknologi Sepuluh Nopember, Surabaya, Indonesia

**Keywords:** *Staphylococcus epidermidis*, O47, genome, biofilm, virulence factors

## Abstract

The skin colonizing coagulase-negative *Staphylococcus epidermidis* causes nosocomial infections and is an important opportunistic and highly adaptable pathogen. To gain more insight into this species, we sequenced the genome of the biofilm positive, methicillin susceptible *S. epidermidis* O47 strain (hereafter O47). This strain belongs to the most frequently isolated sequence type 2. In comparison to the RP62A strain, O47 can be transformed, which makes it a preferred strain for molecular studies. *S. epidermidis* O47’s genome has a single chromosome of about 2.5 million base pairs and no plasmid. Its *oriC* sequence has the same directionality as *S. epidermidis* RP62A, *S. carnosus*, *S. haemolyticus*, *S. saprophyticus* and is inverted in comparison to *Staphylococcus aureus* and *S. epidermidis* ATCC 12228. A phylogenetic analysis based on all *S. epidermidis* genomes currently available at GenBank revealed that O47 is closest related to DAR1907. The genome of O47 contains genes for the typical global regulatory systems known in staphylococci. In addition, it contains most of the genes encoding for the typical virulence factors for *S. epidermidis* but not for *S. aureus* with the exception of a putative hemolysin III. O47 has the typical *S. epidermidis* genetic islands and several mobile genetic elements, which include staphylococcal cassette chromosome (SCC) of about 54 kb length and two prophages φO47A and φO47B. However, its genome has no transposons and the smallest number of insertion sequence (IS) elements compared to the other known *S. epidermidis* genomes. By sequencing and analyzing the genome of O47, we provide the basis for its utilization in genetic and molecular studies of biofilm formation.

## Introduction

*Staphylococcus epidermidis* is a ubiquitous inhabitant of human skin and mucous membranes. Originally, this species was thought to rarely cause infections in normal hosts (Pulverer et al., 1987). However, the number of infections caused by *S. epidermidis* has been steadily increasing with the growing number of immunocompromised patients in hospitals and the widespread medical use of prosthetic and indwelling devices (Christensen et al., 1982a,b, 1994). In the past years, it was found that the major cause of persistent infections is mainly due to the ability to form a biofilm on implant material and tissues. In this regard, almost 80% of the cells involved in biomaterial-related infections are *S. epidermidis*. This phenomenon can be attributed due to easy accessibility of this skin inhabitant to wounds and implants (von Eiff et al., 1999; Götz, 2002).

Therefore, it was not surprising that staphylococci biofilm associated factors have been first investigated in *S. epidermidis*. Already in the early 1980s, electron microscopic studies of polymer devices infected by *S. epidermidis* have shown that multilayered cell clusters of staphylococci are embedded in a thick matrix of a slime substance (Locci et al., 1981; Peters et al., 1982). Later, it was revealed that the slimy material is mainly composed of polysaccharide intercellular adhesin (PIA), a linear ß-1,6-linked glucosaminoglycan (Mack et al., 1996). Almost simultaneously, in the same year, the PIA biosynthesis proteins were found to be encoded by the intracellular adherence (*ica)* operon in the *S. epidermidis* strain O47 (hereafter O47) (Heilmann et al., 1996b). O47 was one of the several *S. epidermidis* isolates from patients with orthopedic implant infection provided by Francois Perdreau-Remington, who was based at University Muenster, Germany at that time (Heilmann et al., 1996a). “O” in this regard stands for orthopedic. We tested these isolates for their suitability in biofilm studies and found that plasmid-free strain O47 is sensitive to antibiotics and transformable, thus making it an ideal strain for molecular studies. Since then, the function of the PIA biosynthesis proteins has been extensively unraveled. IcaA and IcaD (the latter as a helper protein) show an N-acetylglucosaminyltransferase activity (Gerke et al., 1998), and IcaB is a surface-attached protein that is responsible for deacetylation of the poly-N-acetylglucosamine molecule (Vuong et al., 2004). Besides PIA, there are also proteins involved in intercellular aggregation such as the accumulation-associated protein Aap (Hussain et al., 1997); however, Aap acts as an intercellular adhesin only when it is proteolytically processed (Rohde et al., 2005).

Recent advances in molecular biology have allowed researchers to study the molecular basis of biofilm formation. Molecular analysis of the genes involved in biofilm formation revealed that the development of biofilm involves two major steps. The first step is the adherence of bacterial cells to a surface. Once adhered, it can proceed to the second step which is cell aggregation (accumulation phase) (Götz, 2002). A number of adherence factors have been identified that contribute in varying extents to the adherence to various surfaces. Again, one of the first adherence factors, the major autolysin (AtlE), has been described in the strain O47 (Heilmann et al., 1996a, 1997).

In the context of *S. epidermidis*, its biofilm formation appears to be influenced by many factors. Anoxic conditions (Cramton et al., 2001) or pure fermentative growth as in small colony variants of O47 (Al Laham et al., 2007) increased *ica* expression and consequently biofilm formation. Iron limitation enhanced slime production (Deighton and Borland, 1993), and glucose increased and controlled *ica* expression in O47 (Gerke et al., 1998). Furthermore, *ica* expression is influenced by NaCl and ethanol and is also *sigB* dependent in *S. epidermidis* 1457 (Knobloch et al., 2001). Besides, a novel mechanism of phase variation of the *ica* operon has been described based on alternating insertion and excision of the insertion sequence element IS256 (Ziebuhr et al., 1999). As for *ica* genes, there are several lines of evidence indicating its crucial role in the infection by *S. epidermidis*. For example, *ica*-positive *S. epidermidis* strains are prevalent in blood culture strains and mucosal isolates (Ziebuhr et al., 1997). Additionally, the pathogenesis of intravascular catheter-associated infection in a rat model was increased in wild type *S. epidermidis* compared to its corresponding mutants (Fey et al., 1999; Rupp et al., 2001). With the discovery of the *ica* genes in *Staphylococcus aureus* (Cramton et al., 1999), the focus of biofilm studies was shifted to *S. aureus* where the same as well as additional adherence factors have been identified.

However, *S. epidermidis* is nowadays regarded as the most frequent cause of nosocomial infections, at a rate as high as its notorious cousin *S. aureus* (National Nosocomial Infections Surveillance, 2004). Although infections caused by *S. epidermidis* seldom turn into potentially life-threatening conditions, they pose a serious burden for the general health system because of their high occurrences and difficulty in treating these infections. Despite this prevalence, *S. epidermidis* remains under-represented in the scientific literature, especially lagging behind the research of its more virulent cousin *S. aureus* (Sabate Bresco et al., 2017). In this respect, the first genome-based analysis of virulence genes was carried out in *S. epidermidis* ATCC 12228 (hereafter, ATCC 12228), a non-biofilm-forming, non-infection associated strain used for the detection of residual antibiotics in food products (Zhang et al., 2003), followed by *S. epidermidis* RP62A (ATCC 35984) (hereafter, RP62A), a methicillin-resistant biofilm isolate (Gill et al., 2005). RP62A is a slime-producing strain isolated between 1979 and 1980 during the outbreak of intravascular catheter-associated sepsis in Memphis, Tennessee (Christensen et al., 1982b). As such, the first molecular-based biofilm studies have been carried out in RP62A (Schumacher-Perdreau et al., 1994).

That being said, we previously discovered that RP62A has a disadvantage in the studies of molecular basis of biofilm formation and infection as it is resistant to DNA transformation and its plasmid (pEpi62) encodes resistance to erythromycin, kanamycin, streptomycin, and penicillin (Schumacher-Perdreau et al., 1994). Furthermore, we were unsuccessful in curing the 29-kb pEpi62. Therefore, we used O47 for molecular analysis, which was quite similar to RP62A but had less biofilm-forming capacity (Heilmann et al., 1996a). In contrast to RP62A, O47 could be transformed, was sensitive to antibiotics (erythromycin, kanamycin, chloramphenicol, novobiocin and tetracycline), carries no plasmid and hence is more appropriate for transposon mutagenesis (Heilmann et al., 1996a). For these reasons, O47 was chosen for further genetic analyses of biofilm formation and virulence studies. O47 is functionally *agr*-negative, and hardly produces delta-toxin and other phenol-soluble modulins (PSMs) which are *agr*-controlled (Vuong et al., 2003; Cheung et al., 2010). Because of the pioneering studies on biofilm formation and virulence in O47, we analyzed its genome and compared with other known staphylococcal genomes. Here, we highlight a few of the outcomes.

## Results

### General Features of the Genome

**FIGURE 1:**
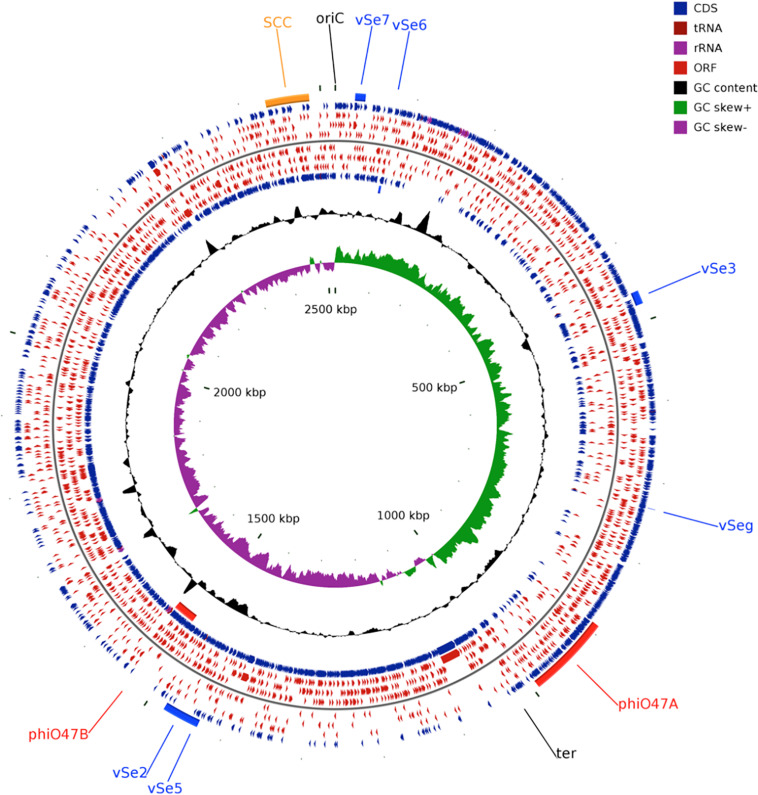
Circular genome plot of *S. epidermidis* O47. From outer to inner circle: ORFs of the forward strand, ORFs in the 3 different reading frames of the forward strand, ORFs of the reverse strand, ORFs in the 3 different reading frames of the reverse strand, G+C content, G+C skew. The regions containing the Staphylococcal Cassette Chromosome (SCC, orange), the putative prophages ϕO47A, ϕO47B (red), and the genomic islands νSe2, νSeγ, νSe3, νSe5, νSe6, and νSe7 (blue) are highlighted. The plot was generated using CGView (Stothard and Wishart, 2005).

**FIGURE 2:**
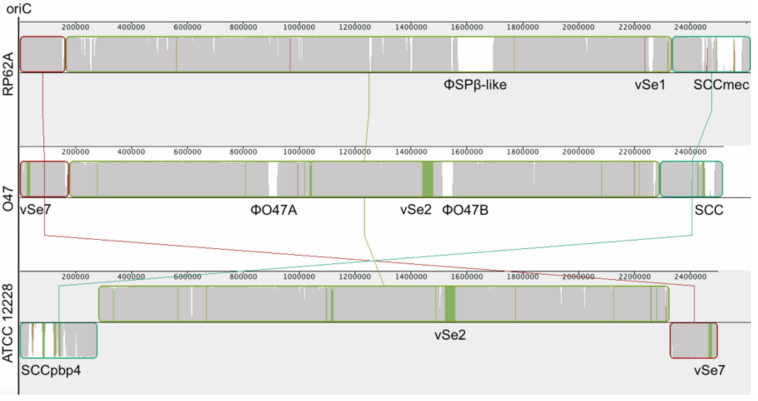
Mauve (Darling et al., 2010) alignment of *S. epidermidis* RP62A (top), O47 (middle) and ATCC 12228 (bottom). In order to accentuate the collinearity between RP62A and O47 genomes, the latter has been inverted for the alignment. The blocks are genomic regions which are aligned to a part of another genome. They are free from genomic rearrangement and lie above the center line if the region is in forward orientation relative to the reference sequence (RP62A at topmost) or down the center line for reverse complement orientation. The similarity profile is displayed in the blocks. White areas are regions not aligned to other genomes and colored areas are conserved among two genomes. The Staphylococcal Cassette Chromosomes (SCC), ϕSPβ, the putative prophages ϕO47A and ϕO47B and the genomic islands νSe1, νSe2, and νSe7 are highlighted.

The whole genome of O47 was *de novo* sequenced using a pyrosequencing approach by the Göttingen Genomics Laboratory at the Institute of Microbiology and Genetics, Georg-August University Göttingen. The assembly of the reads resulted in one contig implying that *S. epidermidis* O47 contains one chromosome and no plasmids. The genome length of O47 is 2,518,182 bp (Figure 1 and Table 1). With 2,408 predicted protein coding genes, it has less proteins than ATCC 12228 and RP62A. Of those in O47, 2035 are CDS with known product and the rest (373) are hypothetical proteins with unknown function. The coding density of 83.3% is similar to ATCC 12228. The coding regions of O47 have a GC content of 32.9%, which is comparable to the other *S. epidermidis* strains. The GC skew of the O47 genome is asymmetrical, which was also observed for ATCC 12228, RP62A, and other coagulase-negative species. The *oriC* of O47 is inverted compared to *S. aureus* and *S. epidermidis* ATCC 12228. Based on our previous study, we observe that O47 shares this feature with the genomes of *S. carnosus, S. haemolyticus, S. saprophyticus and S. epidermidis* RP62A (Rosenstein and Götz, 2013). This inversion is shown in a comparative alignment of the genomes of *S. epidermidis* O47, RP62A and ATCC12228 in Figure 2. Regarding the asymmetrical inversion observed in the genome of O47 related to ATCC 12228, we have reported this observation between *S. epidermidis* strains in our previous study (Rosenstein and Götz, 2013). However, the reason for these differences is unknown. We postulated that it could have arisen from an inversion event during staphylococcal evolution that consisted of the origin of replication in a balanced ancestor genome. Such observation can be seen between the genomes of RP62A and 12228.

**TABLE 1:** General genomic features of the *S. epidermidis* O47 genome in comparison to other staphylococci.

**Strain**	**Size (bp)**	**No. CDS**		**Coding density (%)**	**GC content (%)**		**No. tRNA genes**	**No. rRNA**	
		**Total**	**Hypothetical**		**Total**	**Coding**		**Genes**	**Operons**
***S. epidermidis***									
O47	2 518 182	2 408	373	83.3	32.1	32.9	61	19	5
RP62A	2 616 530	2 542	424	82.6	32.2	32.9	61	19	5
ATCC 12228	2 499 279	2 364	277	83.5	32.1	32.9	60	16	5
***S. aureus***									
N315	2 814 816	2 724	341	83.5	32.8	33.6	62	16	5
USA300	2 872 769	2 863	453	82,1	32.8	33.5	53	16	5
NCTC 8325	2 821 361	2 767	1 510	85,1	32.9	33.5	61	16	5
***S. haemolyticus***									
JCSC1435	2 685 015	2 559	284	86.0	32.8	33.4	59	16	5
***S. saprophyticus***									
ATCC 15305	2 516 575	2 471	215	83.7	33.2	34.0	61	20	5
***S. carnosus***									
TM300	2 566 424	2 441	312	85.8	34.6	35.3	60	16	5

### *S. epidermidis* Phylogenetic Analysis

**FIGURE 3:**
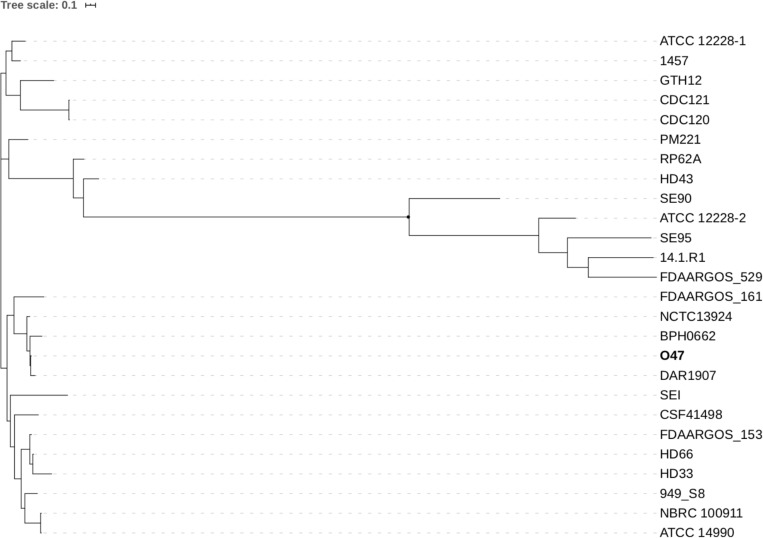
Phylogenetic tree of 25 fully assembled *S. epidermidis* strains available on GenBank and *S. epidermidis* O47 computed by Parsnp.

For the computation of the phylogenetic tree, all 25 available fully assembled genomes of the species *S. epidermidis* were analyzed in an k-mer based approach using Parsnp (Treangen et al., 2014). Parsnp computes a core-genome alignment, and a phylogenetic tree based on the core-genome single nucleotide polymorphisms (SNPs). According to the resulting phylogram (Figure 3), *S. epidermidis* O47 is closest related to DAR1907 and BPH0662. The phylogenetic tree based only on a set of housekeeping genes (*arcC, aroE, gtr, mutS, pyrR, tpiA, yqiL*) used for multilocus sequence typing (Thomas et al., 2007) shows the same strains to be closest related to O47 (Supplementary Figure S1). However, both trees do not match completely.

### Noncoding RNAs

We searched for bacterial ncRNAs (noncoding RNAs) in the *S. epidermidis* strains O47, RP62A, and ATCC 12228 from the Rfam 10.1 database (Supplementary Table S1). We found 38 ncRNA families conserved in the three *S. epidermidis* strains. In nine of these families, we could observe a small difference in the number of detected ncRNAs while the remaining 30 families showed the same number of ncRNAs. In all strains, we found tmRNA, ctRNA, RNAIII, RsaA, RsaD, RsaE, RsaH, and RsaOG.

### Repetitive Elements

Clustered regularly interspaced short palindromic repeats (CRISPRs) contribute to prevent conjugation and plasmid transformation (Marraffini and Sontheimer, 2008). We used the CRISPRfinder tool (Grissa et al., 2007a) to find CRISPR elements in *S. epidermidis* O47. We found four candidates for O47 (Supplementary Table S2) and *S. epidermidis* 1457. According to CRISPRdb (Grissa et al., 2007b), ATCC 12228 contains one candidate, RP62A contains one CRISPR element and one candidate. Like ATCC 12228, the genome of *S. epidermidis* O47 lacks CRISPR-associated genes (*cas1*, *cas2*, and *cas6*) and *cas* subtype *M. tuberculosis* genes (*csm1* - *csm6*) which are present in RP62A (Marraffini and Sontheimer, 2008). The signature sequence for another kind of repeats, the *S. aureus* repeat (STAR) element (Cramton et al., 2000), is present in ten loci of the O47 genome (Supplementary Table S3). The same magnitude of occurrences can be observed for the strains ATCC 12228 and RP62A with nine and eight times, respectively.

### Truncated and Fragmented Genes

We identified ten genes whose coding sequence is split into two ORFs and seven truncated genes (Table 2). These fragmented genes are two hypothetical proteins, the fibrinogen binding protein gene *sdrG*, the glucose-6-phosphate 1-dehydrogenase *zwf*, the plasmid recombination enzyme gene *pre*, a manganese transport protein gene, the accessory gene regulator C *agrC*, the lipase *gehC*, and a two-component sensor histidine kinase. The eight truncated genes of O47 in comparison to the RefSeq annotations of ATCC 12228 and RP62A are five hypothetical proteins, the arsenate reductase *arsC*, and the metallothiol transferase *fosB*. We verified the fragmentation or truncation of the genes *sdrG*, *zwf*, *agrC*, *gehC* and *fosB* by Sanger-Resequencing.

**TABLE 2:** Truncated and fragmented genes in *S. epidermidis* O47.

**Gene**	**Product**	**Locus**
	fragmented hypothetical	FHQ17_10340
	fragmented hypothetical	FHQ17_06240
*sdrG*	fragmented fibrinogen binding protein	FHQ17_112850
*zwf’*	fragmented glucose-6-phosphate 1-dehydrogenase	FHQ17_09435
*zwf”*		FHQ17_09425
*pre*	fragmented plasmid recombination enzyme	FHQ17_09285
	fragmented manganese transport protein	FHQ17_08925
*agrC*	fragmented accessory gene regulator C	FHQ17_04215
*gehC*	fragmented lipase	FHQ17_01050
	fragmented putative lipoprotein	FHQ17_00615
	fragmented two-component sensor histidine kinase	FHQ17_00540
		FHQ17_00535
	truncated hypothetical	FHQ17_12305
		FHQ17_12280
		FHQ17_11070
		FHQ17_05450
		FHQ17_00515
*arsC‘*	truncated arsenate reductase	FHQ17_1127
*fosB‘*	truncated metallothiol transferase	FHQ17_00805

### *S. epidermidis* O47 Specific Genes

We found 187 genes in O47 but not in ATCC 12228 and RP62A when comparing the RefSeq annotations. These genes include the 61 genes of the staphylococcal cassette chromosome (SCC) and the genes of the putative prophages ϕO47A (48 genes) and ϕO47B (47 genes). The remaining genes are 15 transposases and 16 hypothetical genes.

### *S. epidermidis* Species Specific Genes

Next, we analyzed which genes are specific for the *S. epidermidis* species. For this, we searched for genes found only in *S. epidermidis* O47, ATCC 12228, and RP62A and not found in *S. aureus* N315, USA300 and NCTC 8325, *S. haemolyticus* JCSC1435, *S. saprophyticus* ATCC 15305, *S. carnosus* TM300, *S. lugdunensis* HKU09-01, *S. pseudintermedius* HKU10-03 or in the draft annotations of *S. warneri* L37603, *S. capitis* SK14, *S. hominis* SK119 and *S. lugdunensis* M23590. We identified 46 genes where 16 of these are not hypothetical genes such as cell wall located protein genes, *ebh*, *epbS* and *gldA* (Table 3).

**TABLE 3:** *S. epidermidis* species genes.

**Gene**	**Product**	**Locus**
	putative lipoprotein	FHQ17_10710
		FHQ17_10095
		FHQ17_00490
	cell wall surface anchor family protein	FHQ17_08795
		FHQ17_05655
		FHQ17_04260
	surface lipoprotein-related protein	FHQ17_07780
		FHQ17_05475
	putative transposase	FHQ17_09440
*psm*β*3*	phenol soluble modulin beta 3	FHQ17_08695
*ebh*	extracellular matrix binding protein	FHQ17_07125
*epbS*	elastin binding protein	FHQ17_06935
	Transposase	FHQ17_03145
*gldA*	glycerol dehydrogenase	FHQ17_00820
	tributyrin esterase	FHQ17_00780
	K05846 osmoprotectant transport system permease protein	FHQ17_00765
*mqo2*	malate:quinone oxidoreductase	FHQ17_00510

### Mobile Genetic Elements

Like other staphylococci, *S. epidermidis* contains several mobile genetic elements (Table 4). Besides the housekeeping recombinases (*recA, recD, recF, recG, recJ, recN, recO, recQ, recR, recU, recX, xerC, xerD*) for DNA replication and repair, the O47 genome contains some site-specific recombinases which can participate in mobile genetic elements. The contained cassette chromosome recombinase ccrC is part of the SCC and the Sin recombinase (FHQ17_09405) regulates strand exchange in *S. aureus* (Rowland et al., 2002). The latter is also present in ATCC 12228 but currently is not annotated in RP62A. Furthermore, a truncated plasmid recombination enzyme containing a stop codon leading to the frame +2 ORFs *pre* (FHQ17_09285). This suggests that the protein could be truncated by the integration of a plasmid. Besides, we found nine integrase and 44 transposase genes where 16 of these are IS1272 transposase or truncated IS1272 transposase genes although the genome is absent of the IS1272 element.

**TABLE 4:** Mobile elements of *S. epidermidis* in comparison to other staphylococci.

**Strain**	**IS elements**	**Transposons**	**Prophages**	**SCC**	**Genomic island**
***S. epidermidis***					
O47	15	−	2	1	6
RP62A	17	4	1	1	6
ATCC 12228	18	−	−	2	7
***S. aureus***					
N315	11	5	1	1	3
USA300	6	2	2	1	3
NCTC 8325	2	−	3	−	2
***S. haemolyticus***					
JCSC1435	82	2	2	1	5
***S. saprophyticus***					
ATCC 15305	2	−	−	2	1
***S. carnosus***					
TM300	−	−	1	−	1

The genome of O47 contains two putative prophages which were named ϕO47A (FHQ17_08005-FHQ17_07785) and ϕO47B (FHQ17_04820-FHQ17_05045) (Figures 1, 2). They are 33kb and 37kb in size, respectively, and thus belong to the *Staphylococcus* class II *Siphoviridae* phages according to (Deghorain and Van Melderen, 2012). Both sites contain integrase, lambda repressor like, sigma-like factor, phage terminase, phage portal protein, tail protein, and holin genes. According to PHASTER analysis (Arndt et al., 2016), the 33 kb prophage (ϕO47A) is incomplete and the other 37 kb prophage (ϕO47B) is classified as complete.

We used megablast (Zhang et al., 2000) on a database of IS elements of Staphylococci provided by Siguier et al. (2006) and identified 15 IS elements in O47 (Supplementary Table S4). The IS6-family IS431mec-like elements are all located in the SCC (Figure 1 and Supplementary Figure S2). Like ATCC 12228, the genome of O47 lacks transposons in contrast to RP62A which contains the transposons Tn554 and Tn4001.

Several genomic islands were identified for *S. epidermidis* (Supplementary Table S5). νSe1 is found only in RP62A except for the universal stress protein family gene SERP2220 which has an ortholog in ATCC 12228. The genome of RP62A, on the other hand, lacks the island νSe2. The islands νSeγ, νSe3, νSe5 and νSe6 can be found in all three of these genomes. Except for the lipoprotein-related protein gene FHQ17_07780, νSe4 is absent in O47. However, ϕO47A can be found near this locus. An integrase gene is absent in the islands νSe1 and νSeγ. The integrase for νSe6 is fragmented in O47 and ATCC 12228. None of the RP62A islands contain an integrase gene.

The multiple sequence alignment (Figure 2) shows another region named νSe7 which is contained in O47 (Bases 24,528 - 36,133) and ATCC 12228 (Bases 2,464,131 - 2,485,736) but not in RP62A. This region contains a total of eight genes. FHQ17_12320 is the *sdrF* gene and the other genes are a glucosyltransferase gene (FHQ17_12310), an acetyltransferase gene (FHQ17_12305), and an ABC transporter gene (FHQ17_12285). The other four genes are hypothetical ones.

### Staphylococcal Cassette Chromosome (SCC)

The genome of O47 contains a 54kb allotype-5 SCC non-mec element (Supplementary Figure S2). Composite SCC elements are usually separated by conserved direct repeats (DR) (Urushibara et al., 2020). We checked the O47 SCC for such elements but we found only two conserved direct repeats: one is highly similar to the DR-6 (found also in *S. aureus* and *S. epidermidis* ATCC12228) and the other one to DR-2 (also found in the aforementioned *Staphylococcus* species). We also found two other direct repeats with a length of 18 bases each (which seems to be the usual length of these repeats) within the SCC but these are not described elsewhere. Therefore, we have no clear indications for a composite SCC as described in the literature. The SCC is flanked by the classical SCC-specific terminal repeats and contains a cassette chromosome recombinase C7 (*ccrC* at locus FHQ17_00210) with an identity of 100% to the *S. epidermidis* ccrC7 gene (accession ABP68833). We found four IS elements in the SCC: two IS431mec-like elements flank the mercury resistance cluster (Laddaga et al., 1987) while two IS431mec-like elements are located between *ccrC* and *orfX* flanking a conserved hypothetical protein and a probable manganese transport protein (*mntH*). Besides these, the SCC element also contains the arsenical resistance cluster, a multicopper oxidase (*mco*), a copper transporting ATPase (*copB*), the restriction modification system (*hsd*) and the DNA repair protein gene *radC*.

### Metabolic Pathways of O47 in Comparison to Other *S. epidermidis* Strains

The KEGG Automatic Annotation Server (KAAS), Moriya et al. (2007) was used to identify the functional properties and biological roles of the O47, ATCC 12228, and RP62A genes and the differences between these strains were examined. Regarding the metabolic KEGG pathways, O47 and ATCC 12228 contain genes which are missing or nonfunctional in RP62A. One of these is a glutamate synthase (large chain) gene *gltB* (K00265) whose product is involved in the nitrogen (ko00910) and the alanine, aspartate and glutamate metabolism (ko00250). In RP62A, *gltB* (SERP0108) is a pseudogene since it contains a frame shift. Another frame shift can be observed for the RP62A pseudogene *pabB* (SERP0375). The *pabB* product and the product of the gene *pabC*, which is missing in RP62A in contrast to the other two strains, participate in the folate biosynthesis (ko00790). We found that the genes *pbp4* (K07258) and a hypothetical *vanY* (K07260), which are involved in peptidoglycan biosynthesis (ko00550), are specific for ATCC 12228 and thus are missing in O47 and RP62A.

On the other hand, RP62A contains genes which are absent in the other two strains. Besides the methicillin resistance genes *mecR1* (K02547), *mecI* (K02546), and *mecA* (K02545), RP62A contains a DNA (cytosine-5-)-methyltransferase gene (K00558) participating in the cysteine and methionine metabolism (ko00270). In addition, RP62A is specific for a gene (K00680) whose product is involved in the tyrosine metabolism (ko00350), benzoate degradation (ko00362), naphthalene degradation (ko00626), aminobenzoate degradation (ko00627), ethylbenzene degradation (ko00642), and limonene and pinene degradation (ko00903).

Recently, O47 has been reported to be able to produce trace amines from aromatic amino acids. We then characterized a *sadA* gene, which was first described in *S. pseudintermedius* ED99 and encodes an aromatic amino acid decarboxylase (Luqman et al., 2018), which is also present in O47 (FHQ17_00300). It has 52% identity with the *sadA* gene in *S. pseudintermedius* ED99 and 99% identity with a gene encoding pyridoxal-dependent decarboxylase located in locus SE0112 in ATCC 12228 genome. However, we did not find the *sadA* homolog in RP62A.

### Non-metabolic Pathways of O47 in Comparison to Other *S. epidermidis* Strains

Regarding the non-metabolic KEGG pathways, we found *uhpT* (K07784), which is involved in the glucose-6-P uptake and assigned to the two-component system pathway (ko02020), present in O47 and ATCC 12228 but not in RP62A. According to the KAAS, none of these *S. epidermidis* strains contain the other glucose-6-P uptake genes *uhpA* (K07686), *uhpB* (K07675), or *uhpC* (K07783).

Although all of the three *S. epidermidis* strains contain two ATP-binding protein genes (K09687) of the antibiotic transport system in the ABC transporters pathway (ko2010), only ATCC 12228 has the permease protein gene (K09686). For RP62A, the kdp operon, a two component system (ko02020) and in *E. coli* an inducible high-affinity K^+^ transporter (Altendorf et al., 1994), is specific. The *kdpF* gene (K01545) of this operon, however, is not present in RP62A. Apart from that, RP62A contains an ATP dependent DNA ligase (K01971) which is involved in base excision repair (ko03410), nucleotide excision repair (ko03420), mismatch repair (ko03430), and non-homologous end-joining (ko03450).

### Global Regulatory Systems

The typical global regulatory systems known in staphylococci are involved in cell wall biosynthesis, adhesion, biofilm formation, autolysis, secretion and regulation of exoproteins, and virulence factor expression. Except for *sarS*, *sarT* and *mepRABC*, orthologs for these systems exist in O47 with a high similarity (≥ 98% identity in all cases) to other *S. epidermidis* strains (Table 5). This includes the *aps* system which is equivalent to the *graRS* system of *S. aureus* and belongs to a resistance mechanism to antimicrobial peptides (Herbert et al., 2007; Li et al., 2007; Meehl et al., 2007). In O47, the *agr* system is likely to be nonfunctional since a stop codon in the coding sequence of *agrC* leads to the ORFs *agrC’* (frame +2) and *agrC”* (frame +3). A similar observation for *agrC* was made in *S. carnosus* TM300 (Rosenstein et al., 2009).

**TABLE 5:** Global regulatory systems of *S. epidermidis* O47 and their homologs.

**Gene**	**Locus tag**	**Best hit (strain/accession)**	**Function**	**References**
*agrB*	FHQ17_04225	BCM-HMP0060/ZP_04824327	required for a series of secreted exoproteins	Peng et al., 1988
*agrD*	FHQ17_04220	ATCC 12228/NP_765191		
*agrC*	FHQ17_04215	BCM-HMP0060/ZP_04824325		
*agrA*	FHQ17_04210	ATCC 12228NP_765193		
*arlR*	FHQ17_07265	ATCC 12228/NP_764654	involved in adhesion, autolysis, and extracellular proteolytic activity	Fournier and Hooper, 2000
*arlS*	FHQ17_07270	ATCC 12228/NP_764655		
*apsX*	FHQ17_10815	ATCC 12228/NP_763981	controlling resistance mechanisms to antimicrobial peptides	Li et al., 2007
*apsR*	FHQ17_10810	ATCC 12228/NP_763982		
*apsS*	FHQ17_10805	ATCC 12228/NP_763983		
*lytR*	FHQ17_02300	RP62A/YP_189581	control the rate of autolysis	Brunskill and Bayles, 1996
*lytS*	HQ17_02305	BCM-HMP0060/ZP_04826339		
*mgrA*	FHQ17_10660	ATCC 12228/NP_764012	regulator of autolysis	Ingavale et al., 2005
*saeR*	FHQ17_10550	ATCC 12228/NP_764033		
*saeS*	FHQ17_10555	ATCC 12228/NP_764034		
*rot*	FHQ17_05625	RP62A/YP_188894	regulator of virulence factor expression	McNamara et al., 2000
*sarA*	FHQ17_11000	ATCC 12228/NP_763945	involved in the global regulation of exoproteins	Cheung et al., 1992
*sarR*	FHQ17_03000	ATCC 12228/NP_765423		
*sarV*	FHQ17_03140	ATCC 12228/NP_765395		
*sarX*	FHQ17_10765	BMC-HMP0060/ZP_04824566		
*sarY*	FHQ17_02990	ATCC 12228/NP_765425		
*sarZ*	FHQ17_02505	ATCC 12228/NP_765522		
*sigB*	FHQ17_04040	ATCC 12228/NP_765223	alternate sigma factor	Wu et al., 1996
*ssrA*	FHQ17_06900	RP62A/YP_188631	regulation of virulence factors	Yarwood et al., 2001
*ssrB*	FHQ17_06905	ATCC 12228/NP_764731		
*spxA*	FHQ17_09530	ATCC 12228/NP_764241	impacting stress tolerance and biofilm formation	Pamp et al., 2006
*tcaR*	FHQ17_02655	ATCC 12228/NP_765492	involved in cell wall synthesis	Brandenberger et al., 2000
*tcaA*	FHQ17_02660	ATCC 12228/NP_765491		
*tcaB*	FHQ17_02665	ATCC 12228/NP_765490		
*vraR*	FHQ17_04555	ATCC 12228/NP_765124	regulator of cell wall peptidoglycan synthesis	Kuroda et al., 2003
*vraS*	FHQ17_04550	ATCC 12228/NP_765125		
*yycF*	FHQ17_00110	ATCC 12228/NP_763573		
*yycG*	FHQ17_00115	ATCC 12228/NP_763574		
*yycH*	FHQ17_00120	W23144/ZP_04797681		
*yycI*	FHQ17_00125	ATCC 12228/NP_763576		
*yycJ*	FHQ17_00130	ATCC 12228/NP_763577		
*luxS*	FHQ17_03720	ATCC 12228/NP_765287	repressed biofilm formation through a cell-cell signaling mechanism	Xu et al., 2006

### *S. epidermidis* Virulence Factors in *S. epidermidis* O47

*Staphylococcus epidermidis* genes are involved in biofilm formation, lysozyme and antimicrobial protein (AMP) resistance, toxin production and iron uptake. In O47, most genes for the typical *S. epidermidis* virulence factors according to an overview by Otto (2009) are present (Supplementary Table S6). All these genes have a query coverage > 99% except for *sdrH* which is a consequence of a decreased number of Asp-Ser repeats in the SdrH amino acid sequence. For all genes, the identity is ≥ 98%. The genes for the staphyloferrin A biosynthesis proteins were annotated using the primer sequence provided in Cotton et al. (2009). While the peptide sequence of the annotated phenol soluble modulin (PSM) genes corresponds exactly to these observed by Yao et al. (2005), we did not annotate related sequences.

**FIGURE 4:**
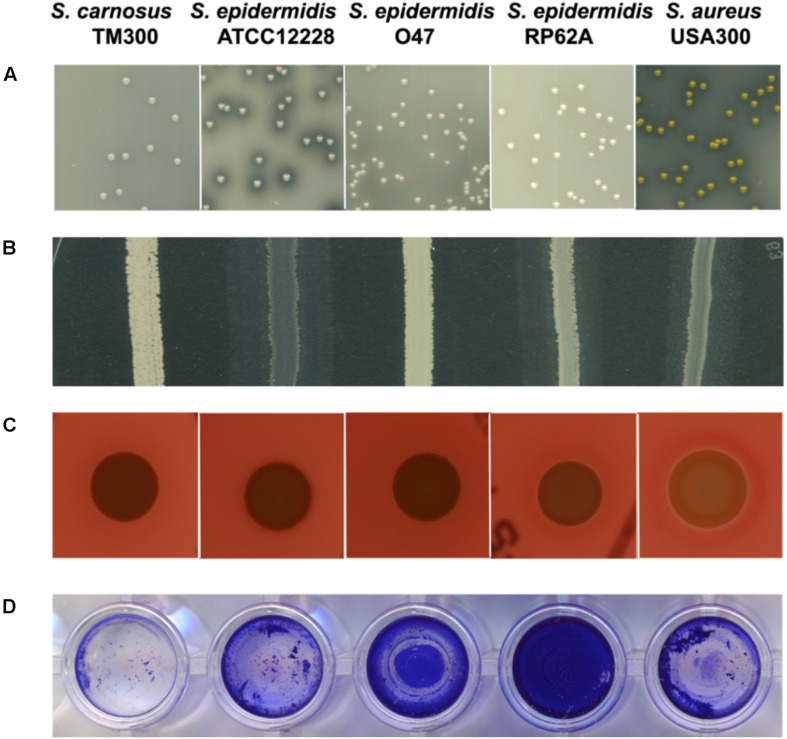
Comparison of characteristics associated with pathogenicity and/or virulence factors among selected *S. epidermidi*s strains. From left: *S. carnosus* TM300, *S. epidermidis* ATCC 12228, *S. epidermidis* O47, *S. epidermidis* RP62A and *S. aureus* USA300. The general virulence factors (in brackets) caused the following characteristics: **(A)** proteolysis (protease) **(B)** lipolysis (lipase) **(C)** hemolysis (hemolysin) **(D)** biofilm formation (accumulation associated protein Aap). *S. epidermidis* O47 showed no protease, lipase and hemolysin activity and moderate biofilm formation. Detailed information on the generation of these results are described in the Methods section. All tests were performed in three independent biological replicates.

As with ATCC 12228 and RP62A, the capsule biosynthesis gene *capD* is missing in O47. Furthermore, the O47 strain lacks the biofilm associated protein gene *bap* which is also absent in ATCC 12228. To evaluate the effect of these missing genes in O47, we carried out a biofilm assay. The biofilm formation in O47 was moderate in comparison to ATCC 12228, which showed no biofilm formation, and RP62A which showed more pronounced biofilm formation (Figure 4).

Indeed, in O47 the virulence factor genes for the lipase GehC and the fibrinogen binding protein SdrG are fragmented in *S. epidermidis* O47 and might be nonfunctional. To examine some of these phenotypes, we performed agar diffusion assays to check for protease and lipase activity. In agreement with the genomic findings, no protease and lipase activity was observed in O47 compared to the other *S. epidermidis* strains (Figure 4).

The *tarIJK* and *tagAHGBXD* clusters which are involved in the teichoic acid biosynthesis (recently reviewed for *S. aureus* by Swoboda et al. (2010), are also present in *S. epidermidis* O47. On the other hand, O47 lacks the genes *tarI’J’L* which are homologous to *tarIJK* as observed in *S. aureus* and were suggested to have the same enzymatic function.

### Peptidoglycan Biosynthesis in *S. epidermidis* O47

The genome of O47 contains the penicillin binding proteins (PBP) 1, 2, and 3 with high identity (≥98%) to other *S. epidermidis* strains. PBPs are involved in the final step of the biosynthesis of peptidoglycan. However, PBP4 is missing or not functional in O47 and RP62A, but is present in ATCC 12228 (Supplementary Table S7).

### O47 Is Penicillin Resistant but Methicillin Sensitive

The penicillin resistance gene and its regulators (*blaIRZ*) are in the genomic sequence next to two hypothetical genes and the Tn554-related transposase gene. The same neighborhood can be observed for ATCC 12228 and RP62A. We determined the minimum inhibitory concentration (MIC) values for penicillin and methicillin in O47 and other strains and found that O47 was resistant to penicillin (MIC > 128 μg/ml) but sensitive to methicillin (MIC < 2 μg/ml) (Supplementary Table S8). *S. aureus* USA300, which is resistant to penicillin and methicillin, was used as a control. As mentioned earlier, we found that metallothiol transferase *fosB* is truncated in O47, which may relate to resistance. Therefore, we also included fosfomycin in the MIC test. However, the MIC of all strains tested were in the range of 1–8 μg/ml which is not conclusive. Besides the penicillin resistance, the genome of O47 contains several other resistances such as an arsenical, a mercuric or an azaleucine resistance (Supplementary Table S9).

### *S. aureus* Virulence Factors in *S. epidermidis* O47

We searched the O47 genome for the typical *S. aureus* virulence factors and found a putative hemolysin III (FHQ17_03540) with high similarity in other *S. epidermidis* strains (identity 100%). However, O47 showed no hemolysis on blood agar (Figure 4). On the other hand, *S. epidermidis* O47 lacks the typical *S. aureus* adhesins, toxins, and invasins (Table 6).

**TABLE 6:** *S. aureus* virulence factors not found in *S. epidermidis* O47.

**Genes**	**Product**
*clfA*	clumping factors
*clfB*	
*sdrC*	Adhesins
*sdrD*	
*sdrE*	
*fnbA*	fibronectin binding protein
*fnbB*	
*cna*	collagen adhesin
*spa*	Immunoglobulin G-binding protein A
*fbpA*	fibrinogen binding protein gene
*ebp*	extracellular adherence protein
*map*	major histocompatibility complex class II analog protein
*vWbp*	Von Willebrand factor
*efb*	extracellular fibrinogen-binding protein
*coa*	Coagulase
*sbi*	immunoglobulin binding protein
*tst*	toxic shock syndrome toxin
*set1-26*	Exotoxins
*entA-B*	Enterotoxins
*entC1-3*	
*entD-H*	
*splA-F*	
*lukE*	Leukotoxins
*lukF*	
*hla*	α-hemolysin
*hlgA-C*	γ-hemolysin
*sak*	Staphylokinase
*eta*	exfoliative toxins
*etb*	
*nuc*	Thermonuclease

## Discussion

A detailed genome analysis for *S. epidermidis* O47 was long overdue. Originally isolated from patient with an orthopedic device associated infection, this strain became important in the 1990s when the molecular basis of biofilm formation in *S. epidermidis* was being elucidated. Prior to this, the phenotype of biofilm formation was mostly studied using a high mucus producer strain RP62A which was isolated in Tennessee, United States (Christensen et al., 1982b). From a molecular biological point of view, RP62A was hardly accessible due to its difficulty in genetic transformation and resistance to antibiotics. In this respect, strong barriers to the exchange of DNA such as clustered regularly interspaced short palindromic repeats (CRISPR) or restriction-modification (RM) systems have reported to prevent horizontal gene transfer events between bacteria (Thomas and Nielsen, 2005; Marraffini and Sontheimer, 2010). In an effort to find a suitable strain that is easy to transform and sensitive to antibiotics, we found O47 to be an ideal candidate for biofilm formation studies in the *S. epidermidis* background (Heilmann et al., 1996a). Our present analyses showed that there are several CRISPR candidates in the genome of O47 but it lacks the CRISPR-associated genes (*cas1*, *cas2*, and *cas6*) and *cas* subtype *M. tuberculosis* genes (*csm1* – *csm6*) which are present in RP62A (Marraffini and Sontheimer, 2008). This probably suggests that the presence of *cas* genes could be the reason RP62A is resilient against DNA transformation. In addition, O47 was also one of the Coagulase-negative staphylococci (CoNS) reference strains used in a study where plasmids from *S. aureus* can be transduced by a unique bacteriophage Φ187; further strengthening the potential of O47 in terms of genetic manipulation (Winstel et al., 2015).

Phylogenetic analysis revealed that O47 is closest related to DAR1907 and BPH0662. As with O47, both are clinical isolates. DAR1907 was a blood isolate from 2007 and the complete genome is available in the NCBI database under the accession number NZ_CP013943. According to NCBI, the sequence was submitted under the title ‘Population structure of hospital adapted *Staphylococcus epidermidis*’ by the University of Mississippi Medical Center in Jan 2020. However, the report was unpublished. Therefore, the associated phenotypic characteristics of this strain, for example its biofilm activity is unknown to date. BPH0662 is a multidrug resistant, hospital-adapted ST2 strain dominant in Australia as reported recently (Lee et al., 2016). BPH0662 represents as the first complete genome of an ST2 *S. epidermidis* strain. In recent years, more genomes of *S. epidermidis* from other sources were reported and analyzed. Apart from those isolated in clinical settings, analysis of a strain G6_2 isolated from the general public environment in London representing ST59 was described (Xu et al., 2018). Comparative genomic analysis of this strain focused more on the antibiotic resistance and its virulence gene arsenal. A very recent study reported three *S. epidermidis* strains isolated from fecal sample of a healthy individual (Garcia-Gutierrez et al., 2020). It was shown that these strains are not phylogenetically distinct from *S. epidermidis* isolated from other human body sites.

On a different note, the typical genes involved virulence factors are related to biofilm formation, antimicrobial resistance, toxin production, iron uptake and lysozyme with the former two genes are linked to the persistence of clinical infections (Schoenfelder et al., 2010). Our analyses found some of the genes in O47 were either fragmented or truncated such as accessory gene regulator C *agrC*, lipase *gehC* and metallothiol transferase *fosB.* Therefore, the truncated gene in O47 could have implications on some of these factors. In comparison to the negative control ATCC 12228 which does not form biofilm, we found that the biofilm formation was moderate in O47 and strong in RP62A. It has been shown that the two-component system ArlRS plays a role in regulating biofilm formation in *ica*- and *aap*-positive clinical *S. epidermidis* isolates and that this is operated via both *ica-* and Aap-dependent pathways (Wu et al., 2014). It could be possible that the activity of ArlRS and/or the non-functional Agr system have an impact on biofilm activity seen in O47. In *S. aureus, agr-*negative genotype boosts biofilm formation because of the upregulation of cell wall bound proteins (Coelho et al., 2008). Accordingly, *agr* might have a similar effect in *S. epidermidis*. With regard to antimicrobial resistance, we found the penicillin resistance gene and its regulators (*blaIRZ*) in the genome of O47. For this purpose, we determined the MIC values and found that O47 was resistant to penicillin but sensitive for methicillin. On the contrary, a study reported that isolates from medical devices were mostly resistant to methicillin (Conlan et al., 2012). Since we also found truncated *fosB* in O47, fosfomycin was included in the MIC test but no concrete interpretation could be made because the strain is sensitive to fosfomycin. As for another truncated gene *gehC*, agar diffusion assay showed no lipase activity, in agreement with the genomic findings. Additionally, we also tested protease but no protease activity was observed in O47, which could be attributed to the defect in *agr* systems as mentioned earlier. Both lipase and protease are involved in staphylococcal pathogenicity. Although O47 lacks the classical *S. aureus* virulence factors, we found a putative hemolysin III in the genome of O47. However, no hemolysis activity on blood agar was found indicating that this putative gene is not functional.

It is not surprising that O47 is *agr*-negative, considering that it was isolated from an orthopedic device infection. In the more pathogenic *S. epidermidis* strains, about half of the isolates from patients with persistent bacteremia or infective endocarditis are *agr*-negative; a situation apparently very similar to that observed in *S. aureus* (Fowler et al., 2004; Moise et al., 2009; Painter et al., 2014). In staphylococcal infections, particularly in the case of chronic conditions, the *agr*-negative phenotype with its overexpression of surface adhesins and down-regulation of toxins, has an advantage over *agr*-positive phenotype. *S. epidermidis* is normally regarded as skin bacteria. However, under certain conditions they lead to persistent infections that are difficult to treat mainly due to the ability to form biofilm at higher rate and to internalize human osteoblasts (Perdreau-Remington et al., 1998; Götz and Peters, 2000; Valour et al., 2013; Post et al., 2017).

Furthermore, O47 has been used as a model strain to study multiple colonization factors, making it and its genome sequence valuable tools. The intracellular adherence (*ica*) genes encoding the polysaccharide intercellular adhesin PIA and also the adherence gene *atlE* (major autolysin) have first been identified by transposon (Tn917) mutagenesis in O47 using the transposon Tn917-carrying plasmid pTV1ts (Heilmann et al., 1996a,b, 1997). Both O47 mutant strains were significantly attenuated in a rat central venous catheter (CVC) infection model (Rupp et al., 2001). In another study, two biofilm mutants of O47 were investigated for hemagglutination activity and it was shown that the *ica* genes contribute to this reaction (Fey et al., 1999). In another first, it was in O47 that the first protein structure with an amidase-like fold, amidase domain AmiE (amidase *S. epidermidis*) with a Gram-positive wall architecture was analyzed (Zoll et al., 2010). O47 was also the model strain used in which the *hemB::ermB* mutant was created to better study the small colony variant (SCV) phenotype (Al Laham et al., 2007). In the context of antimicrobials, O47 was also of the *S. epidermidis* model strains included in the anti-biofilm testing of the lantibiotic gallidermin and rhodomyrtone (Saising et al., 2012, 2014). A more recent example in biofilm studies was the use of O47, along with other *S. epidermidis* strains to analyze the functions of non-coding RNA *rsaE* in biofilm communities (Schoenfelder et al., 2019). In our latest findings, it was reported that a majority of skin *S. epidermidis* isolates express the staphylococcal aromatic amino acid decarboxylase (SadA) which enables the strains to produce trace amines from aromatic amino acids (Luqman et al., 2020). *sadA* was first described in *S. pseudintermedius* ED99 (Luqman et al., 2018) and this gene is also found in O47. The trace amine-producing O47 was shown to accelerate wound healing in mice but not its Δ*sadA* mutant (Luqman et al., 2020). HPLC analysis showed that the trace amines were present in the overnight supernatant of O47 but not in Δ*sadA* mutant, confirming the role of *sadA* in wound healing. All these examples highlight the importance of *S. epidermidis* strain O47 not only in biofilm studies but also in other physiological studies. Such studies have generated an extensive amount of knowledge accumulated over the years. It is about time that this genome sequence is made available to the scientific community as a useful tool for future studies.

## Materials and Methods

### Isolation of High Molecular Weight Genomic DNA From Bacterial Cells

Cells from 10 ml overnight culture were lysed by treatment with lysostaphin in 2 ml lysis buffer (P1 buffer (Qiagen, Hilden) supplemented with 25 μg lysostaphin) for 30 min at 37°C. Preparation of chromosomal DNA from the cell lysate was performed according to the procedure by Marmur (1961).

### Sequencing

The genome of O47 was de novo sequenced in a pyrosequencing approach by the Göttingen Genomics Laboratory (Institute of Microbiology and Genetics, Georg-August University Göttingen). The Genome Sequencer FLX Instrument and Titanium chemistry (Roche Applied Science) was used for DNA nebulization, single-stranded template DNA library preparation and sequencing according to the General FLX Library Protocol of the manufacturer. An assembly of the 261,085 reads (Q40 coverage of 99.93%) with the Roche Newbler Assembler 2.0.1 (454 Life Sciences) obtained 56 contigs. The GAP4 software of the Staden package (Staden et al., 2003) was used for editing of the sequences. Sequencing with ABI 3730xl (Applied Biosystems) of standard PCR and combinatorial multiplex PCR products were used to close remaining sequence gaps. The O47 genome sequence can be accessed in the GenBank database with the accession number CP040883.

### Gene and Function Prediction

The GenDB annotation system (Meyer et al., 2003) was used to predict the ORFs, tRNAs, rRNAs, and to perform a functional ORF annotation. The noncoding RNAs were predicted with nocoRNAc (Herbig and Nieselt, 2011) which uses cmsearch (Nawrocki et al., 2009) and the Rfam 10.1 database (Gardner et al., 2009). The stricter TC (trusted cutoff) thresholds for bacterial Rfam seeds were used for this computation. To identify the functional properties and biological roles of the O47, ATCC 12228 and RP62A genes, we used the KEGG Automatic Annotation Server (KAAS) (Moriya et al., 2007).

### Comparative Genomics

To compare the protein contents, we used Blastp (Altschul et al., 1990) and the reciprocal best hit (RBH) method (Tatusov et al., 1997; Bork et al., 1998). For the computation, we used an *E*-value cut-off of 1e-8 and a coverage threshold of 75%. The coverage threshold was not used for the computation of truncated genes. Genes were considered as truncated if they are in their 3′-end ten or more nucleotides shorter than their orthologous gene in one of the RefSeq annotations of ATCC 12228 or RP62A.

For comparative genomic analysis, we used the strains ATCC 12228 [NC_004461, (Zhang et al., 2003)], RP62A [NC_002976, (Gill et al., 2005)], and the draft *S. epidermidis* sequences of strain W23144 (NZ_ACJC00000000.1), M23864:W2(gray) (NZ_ADMU00000000.1), BCM-HMP0060 (NZ_ACHE00000000.1), and SK135 (NZ_ADEY00000000.1). Additionally, we used the *S. aureus* strains N315 [NC_002745, (Kuroda et al., 2001)], USA300_FPR3757 [NC_007793, (Diep et al., 2006)], NCTC 8325 [NC_007795, (Gillaspy et al., 2006)], and the strains *S. haemolyticus* JCSC1435 [NC_007168, (Takeuchi et al., 2005)], *S. saprophyticus* ATCC 15305 [NC_007350, (Kuroda et al., 2005)], *S. carnosus* TM300 [NC_012121, (Rosenstein et al., 2009)], *S. lugdunensis* HKU09-01 [NC_013893, (Tse et al., 2010)], and *S. pseudintermedius* HKU10-03 [NC_014925, (Tse et al., 2011)]. Furthermore, we used the draft sequences of the strains *S. warneri* L37603 (NZ_ACPZ00000000), *S. capitis* SK14 (NZ_ACFR00000000), *S. hominis* SK119 (NZ_ACLP00000000), and *S. lugdunensis* M23590 (NZ_AEQA00000000).

### Transposons

To identify transposons in the O47 genome, we used Blastn (Zhang et al., 2000) and the sequences of transposons Tn551 (accession number Y13600), Tn552 (X52734), Tn554 (X03216), Tn558 (58577493), Tn559 (302064329), Tn4001 (13383306), Tn4003 (13383306), Tn5404 (L43098.1), Tn5406 (AF186237.2), Tn5801 (289166909), Tn6072 (GU235985.1), and the *S. epidermidis* composite transposon (13383306) as query. We used an E-value cut-off of 1e-8 and a coverage threshold of 90%.

### Phylogenetic Analysis

The phylogenetic analysis was applied to 25 fully assembled strains of the *S. epidermidis* species available on GenBank and *S. epidermidis* O47 (assessed on 9 Aug 2019). Full genomes were used as an input for the tool Parsnp (Treangen et al., 2014) for tree construction. iTol (version 5.5.1) (Letunic and Bork, 2007) was used for tree visualization. The phylogenetic tree based on a set of housekeeping genes (*arcC, aroE, gtr, mutS, pyrR, tpiA, yqiL*) was built on the concatenated gene sequences.

### Repeats

The CRISPR finder tool (Grissa et al., 2007a) was used to find CRISPRs in the O47 genome. Blastn with a word size of 14 was used for the identification of the STAR element signature sequence (Cramton et al., 2000).

### Protease Activity Test by Agar Diffusion Assay

Overnight cultures of staphylococcal strains were adjusted to OD_578_ of 1 and were streaked on the skim milk agar (skim milk powder 2.8%, tryptone 0.5%, yeast extract 0.25%, glucose 0.1%, and agar 1.5% at pH 7). The plates were incubated overnight at 37°C and subsequently stored at 4°C for an additional 24 h. Three independent biological replicates were performed for this assay. Protease activity was observed as visible halo due to the casein degradation.

### Lipase Activity Test by Agar Diffusion Assay

The tryptic soy agar (TSA) plates containing 1% Tween 20 were used to monitor lipase activity of different staphylococcal strains. Overnight cultures of staphylococcal strains were adjusted to OD_578_ of 0.1 and 10 μl was dropped on the Tween 20 containing TSA. The plates were incubated overnight at 37°C and subsequently stored at 4°C for an additional 24 h. Three independent biological replicates were performed for this assay. Lipase activity was observed as a visible halo due to the precipitation of liberated fatty acids.

### Hemolysis Assay

Overnight cultures of staphylococcal strains were adjusted to OD_578_ of 0.1 and 10 μl was dropped on the Columbia sheep blood agar plates (Thermo Scientific). The plates were incubated overnight at 37°C and subsequently stored at 4°C for an additional 24 h. Three independent biological replicates were performed for this assay. Hemolysis activity was indicated by the presence of a visible halo was observed due to the erythrocytes lysis.

### Biofilm Assay

The biofilm assay was performed according to (Saising et al., 2012) with modifications. Overnight cultures of staphylococcal strains grown in TSB with an additional 0.25% glucose were adjusted to OD_578_ of 0.1. 20 μl of the bacterial suspension was added to 180 μl of TSB in wells of a 96 well flat bottom microtiter plate (Greiner Bio-One), resulting in a final OD_578_ of 0.01 which corresponds to approximately 10^6^ CFU/ml. The microtiter plate was incubated at 37°C without agitation for 24 h. After 24 h, the culture supernatant was discarded and the wells were rinsed twice with 200 μl of PBS before being air dried for 30 min. Then, the wells were stain with 200 μl of 01% crystal violet for 30 min and subsequently rinsed with purified water (MilliQ). The plate was air dried again for 30 min and image of the wells was taken with an image scanner (Epson). Three independent biological replicates were performed for this assay.

### MIC Determinations

The MIC values were determined by the microdilution method. *S. epidermidis* ATTC 12228 was used as a non-clinical reference strain for quality control and *S. aureus* USA300, a MRSA strain which is resistant to penicillin and methicillin as another control. Antibiotics used (penicillin and methicillin) were serially diluted (128 μg/ml to 0.25 μg/ml) with Muller Hinton Broth (MHB) (supplemented with 2% NaCl) in 96 well microtiter plates. For fosfomycin, MHB was supplemented with 25 μg/ml of glucose-6-phosphate. Equal volumes of bacterial inoculum from overnight cultures adjusted to the final OD_578_ of 0.05 were added. The microtiter plates were incubated at 37°C with continuous shaking for 18 h. The MIC was determined as the lowest concentration that completely inhibited visible growth of the bacteria. The MIC determinations were performed in three independent biological replicates.

## Conclusion

The *S. epidermidis* O47 genome was sequenced, assembled and analyzed. Its GC content of the coding regions is 32.9%, similar to the other *S. epidermidis* strains and is predicted to have less proteins than ATCC 12228 and RP62A. Apart from the genes of typical staphylococci global regulatory systems, O47 also contains the typical *S. epidermidis* genetic islands and some mobile genetic elements. As for virulence factors, it has most of the genes typical for *S. epidermidis* but not for *S. aureus*, except for a putative hemolysin III but we found no hemolysis activity in O47. We also found that the biofilm formation in O47 is intermediate, compared to a stronger one seen in RP62A. In addition, O47 contains no plasmid and therefore is sensitive to antibiotics, making it preferred strain for transposon mutagenesis. The importance of O47 was highlighted in various studies utilizing this strain in biofilm and other physiology studies. Our genome sequencing results offer a basis for O47 to be utilized as a promising candidate to study the molecular basis of biofilm formation and also virulence studies in *S. epidermidis*.

## Data Availability Statement

The genome sequence of *Staphylococcus epidermidis* O47 has been deposited in NCBI GenBank under the accession number CP040883 and BioProject number PRJNA546513.

## Author Contributions

FG and RR conceived the idea and designed the study. SR, S-HF, SZ, and AL performed the research and analyzed the data with supervision from FG, RR, and KN. FG, RR, and SR wrote the original draft. FG, S-HF, SZ, and KN corrected and critically evaluated the manuscript with input from all authors.

## Conflict of Interest

The authors declare that the research was conducted in the absence of any commercial or financial relationships that could be construed as a potential conflict of interest.

## Abbreviations

Aap: accumulation associated protein
ABC transporter: ATP-binding cassette transporter
AMP: antimicrobial peptides
ATP: Adenosine Triphosphate
CoNS: Coagulase-negative staphylococci
CRISPR: clustered regularly interspaced short palindromic repeats
DNA: Deoxyribonucleic acid
KAAS: Automatic Annotation Server
KEGG: Kyoto Encyclopedia of Genes and Genomes
PIA: polysaccharide intercellular adhesin
PSM: phenol soluble modulin
RBH: reciprocal best hit
RNA: Ribonucleic acid
SCC: Staphylococcal Cassette Chromosome
STAR: *S. aureus* repeats.
