# Supplementary material for: The Genome of Staphylococcus epidermidis O47
Source: Front Microbiol. 2020 Aug 25;11:2061. doi: 10.3389/fmicb.2020.02061 (PMC7477909; doi:10.3389/fmicb.2020.02061)
Supplement: Supplementary file 1 [file Data_Sheet_1.pdf]

Supplementary Information

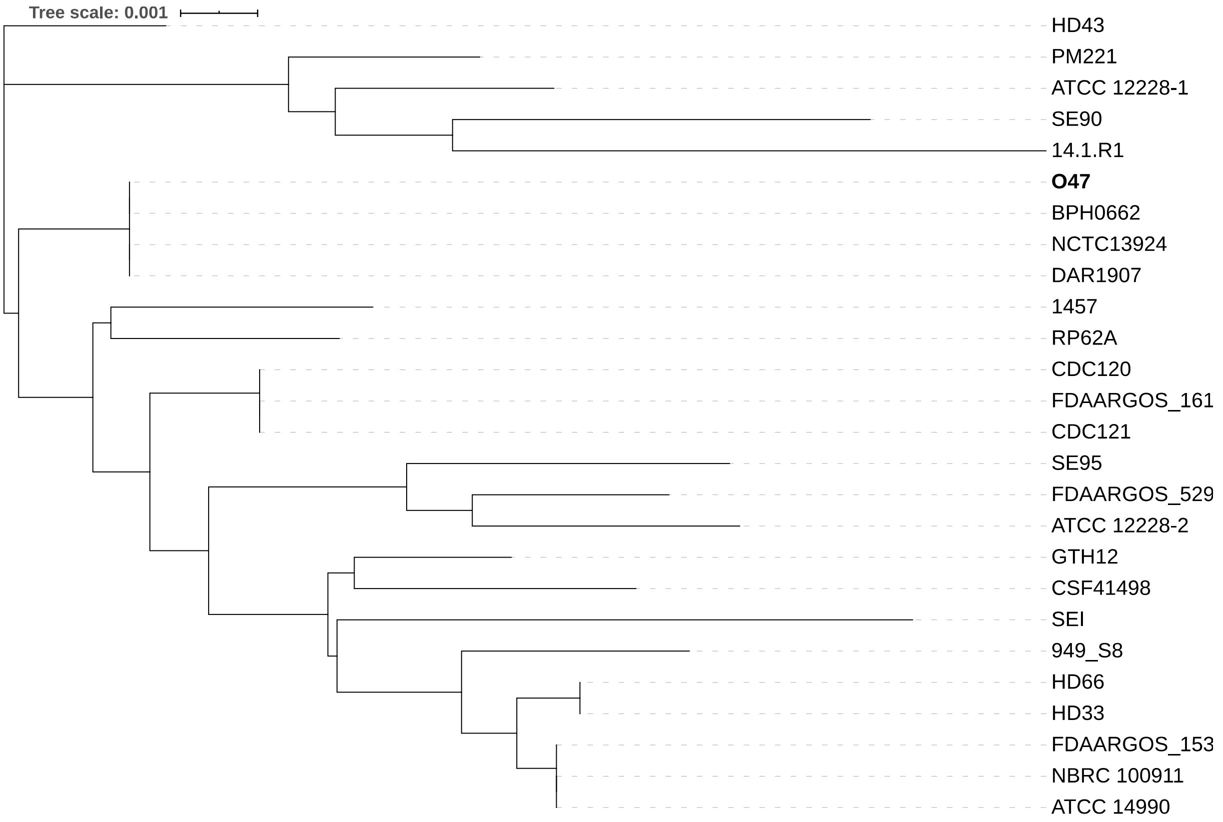

Supplementary Figure 1: Phylogenetic tree based on the housekeeping genes *arcC, aroE, gtr, mutS, pyrR, tpiA,* and *yqiL* of 25 *S. epidermidis* strains available on GenBank and *S. epidermidis* O47.

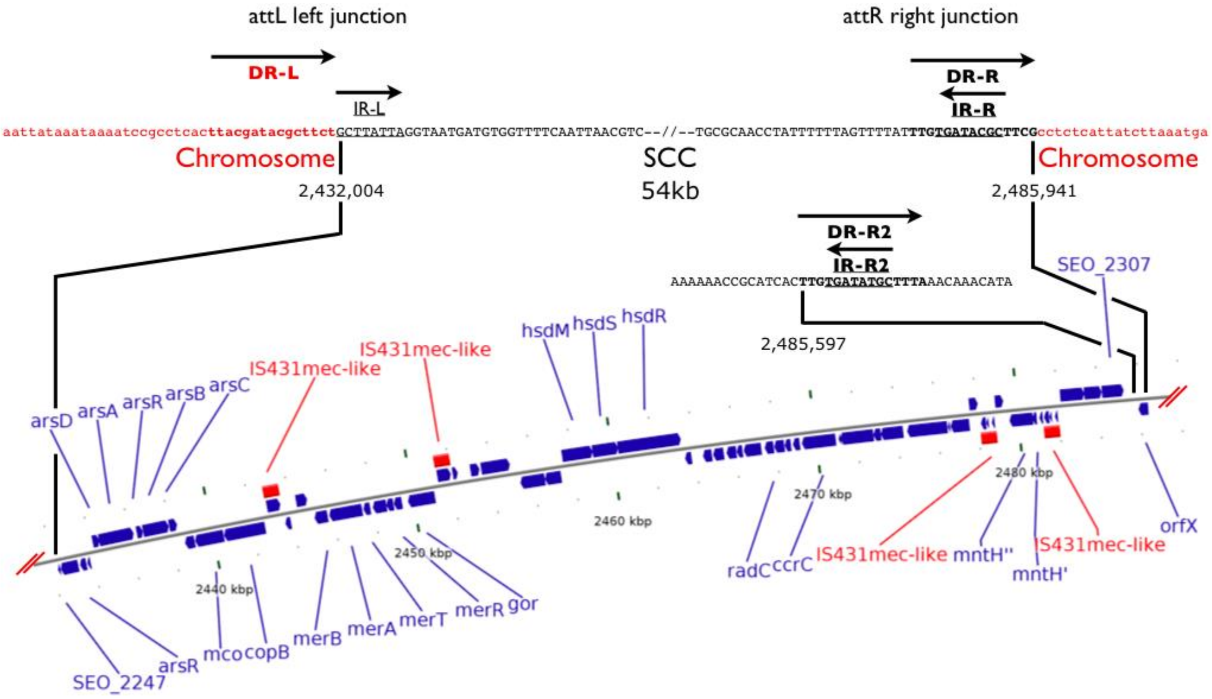

Supplementary Figure 2: The junction sites and the region of the *S. epidermidis* O47 genome showing the Staphylococcal Cassette Chromosome (SCC). The SCC contains four IS431mec-like elements. They flank the mercury resistance cluster and a fragmented manganese transport protein (*mntH‘* and *mntH‘‘*), respectively. Additionally, an arsenical resistance cluster, a multicopper oxidase (*mco*), a copper transporting ATPase (*copB*), a glutathione reductase (*gor)*, the restriction modification system (*hsd*), and the DNA repair protein gene *radC* is located in the SCC. Lower case red letters: part of the *S. epidermidis* O47 Chromosome; capital black letters: part of the SCC; bold letters: direct repeat (DR); underlined letters: inverted repeat (IR). The plot was generated using CGview (Stothard and Wishart, 2005).

Supplementary Table 1: sRNA content in *S. epidermidis*

		No. present in		
**Rfam Seed**	**Description**	**O47**	**RP62A**	**ATCC 12228**
RF00010	Bacterial RNase P class A	1	1	1
RF00011	Bacterial RNase P class B	1	1	1
RF00013	6S / SsrS RNA	1	1	1
RF00023	transfer-messenger RNA	1	1	1
RF00050	FMN riboswitch (RFN element)	2	2	2
RF00059	TPP riboswitch (THI element)	5	5	5
RF00080	yybP-ykoY leader	1	1	1
RF00162	SAM riboswitch (S box leader)	4	4	4
RF00167	Purine riboswitch	1	1	1
RF00168	Lysine riboswitch	2	2	2
RF00177	Bacterial small subunit ribosomal RNA	6	6	5
RF00230	T-box leader	14	14	14
RF00234	glmS glucosamine-6-phosphate activated ribozyme	1	1	1
RF00238	ctRNA	2	1	3
RF00503	RNAIII	1	1	1
RF00504	Glycine riboswitch	1	1	1
RF00515	PyrR binding site	2	2	2
RF00522	PreQ1 riboswitch	1	1	1
RF00555	Ribosomal protein L13 leader	1	1	1
RF00556	Ribosomal protein L19 leader	1	1	1
RF00557	Ribosomal protein L10 leader	1	1	1
RF00558	Ribosomal protein L20 leader	1	1	1
RF00559	Ribosomal protein L21 leader	1	1	1
RF01118	Pseudoknot of the domain G(G12) of 23S ribosomal RNA	6	6	5
RF01405	STnc490k Hfq binding RNA	2	2	3
RF01458	*Listeria* sRNA rli23	1	2	2
RF01691	Bacillus-plasmid RNA	1	1	1
RF01694	Bacteroides-1 RNA	1	1	1
RF01725	SAM-I/IV variant riboswitch	1	1	1
RF01751	potC RNA	1	1	1
RF01764	yjdF RNA	1	3	1
RF01775	RNA *S.aureus* Orsay G	1	1	1
RF01797	Fst antitoxin sRNA	4	6	4
RF01816	RNA Staph. aureus A	1	1	1

RF01819	RNA Staph. aureus D	1	1	1
RF01820	RNA Staph. aureus E	1	1	1
RF01821	RNA Staph. aureus H	1	1	1
RF01854	Bacterial large signal recognition particle RNA	1	1	1
	∑	76	80	77

Supplementary Table 2: *S. epidermidis* CRISPR elements

Start	End	Length	Direct Repeat		Spacers	
			Consensus	Length	No.	Length
S. epidermidis O47 CRISPR candidates (CRISPRfinder)						
332,374	332,464	90	CAACATAGAGAATTTCACCGAGAAATTCAACA	32	1	26
795,622	795,723	101	TCATGTATAAGAAACACTAAATACCTATGTATTAAGTG	38	1	25
803,654	803,749	95	CATGCATAACCAAAAGTACGATTTACTTCGTAAA	34	1	27
2,071,669	2,071,752	83	CAACTTGCCTTGTCCGTGGAATTTC	25	1	33
S. epidermidis ATCC 12228 CRISPR candidate (CRISPRdb)						
587,189	587,284	96	CATAAGTGACACAAGCAATTAAAAATTGCAGTG	33	1	30
S. epidermidis RP62A						
CRIPSR candidate (CRISPRdb)						
1,993,515	1,993,597	83	ATCATAGATAGTTTTGCTTCTGTTT	25	1	33
CRIPSR element (CRISPRdb)						
2,517,620	2,517,868	249	GTTCTCGTCCCCTTTTCTTCGGGGTGGGTATCGATCC	37	3	33
S. epidermidis 1457 CRISPR candidates (CRISPRfinder)						
446,430	446,513	83	GAAATTCTACGGACAAGGCAAGTTG	25	1	34
1,722,487	1,722,551	92	TTATACATGAACCTCAAGCTCATGTGTTC	29	1	35
2,034,010	2,034,073	33	CACTGCAATTTTTAATTGCTTGTGTCACTTATG	33	1	30
2,185,718	2,185,777	32	TGTTGAATTTCCCGGTGAAATTCTCTATGTTG	32	1	27

Supplementary Table 3: *S. epidermidis* STAR elements

Strain	No. STAR elements		Start	End	Sequence
	Total	Sequence			
S. epidermidis					
O47	10	3	276,312	276,299	T**G**TGTTG**G**GGCCC**C**
			1,928,864	1,928,851	
			2,100,125	2,100,138	
		6	57,083	57,070	T**T**TGTTG**G**GGCCC**C**
			57,141	57,128	
			357,412	357,399	
			1,293,199	1,293,212	
			1,293,258	1,293,271	
			2,071,708	2,071,721	
		1	57,198	57,185	T**T**TGTTG**G**GGCCC**A**
ATCC 12228	9	3			T**G**TGTTG**G**GGCCC**C**
		5			T**T**TGTTG**G**GGCCC**C**
		1			T**T**TGTTG**G**GGCCC**A**
RP62A	8	1			T**G**TGTTG**G**GGCCC**C**
		6			T**T**TGTTG**G**GGCCC**C**
		1			T**T**TGTTG**G**GGCCC**A**

S. aureus			
		36	T**G**TGTTG**G**GGCCC**C**
		9	T**G**TGTTG**G**GGCCC**A**
N315	58	3	T**A**TGTTG**G**GGCCC**A**
		6	T**T**TGTTG**G**GGCCC**C**
		4	T**T**TGTTG**G**GGCCC**A**
		21	T**G**TGTTG**G**GGCCC**C**
		5	T**G**TGTTG**G**GGCCC**A**
		3	T**A**TGTTG**G**GGCCC**A**
USA300	42	1	T**A**TGTTG**T**GGCCC**C**
		6	T**T**TGTTG**G**GGCCC**C**
		6	T**T**TGTTG**G**GGCCC**A**
		22	T**G**TGTTG**G**GGCCC**C**
		5	T**G**TGTTG**G**GGCCC**A**
		3	T**A**TGTTG**G**GGCCC**A**
NCTC 8325	42	1	T**A**TGTTG**T**GGCCC**C**
		7	T**T**TGTTG**G**GGCCC**C**
		4	T**T**TGTTG**G**GGCCC**A**
S. carnosus			
TM300	0		

Supplementary Table 4: Family characterization of the 15 IS elements in *S. epidermidis* O47

IS element	Family	Length (Ref)	Start	Stop	Strand
ISSep2	IS110	1564 (1564)	176,242	177,805	+
			382,194	383,757	-
			549,603	551,166	+
			570,617	572,182	-
			1,423,352	1,424,915	+
			1,839,954	1,841,517	-
			1,893,463	1,895,026	+
			2,079,660	2,081,223	-
ISSep3	IS200/IS605	741 (741)	1,241,794	1,242,534	-
			2,217,989	2,218,729	-
IS431mec-like	IS6	790 (790)	2,442,798	2,443,587	+
			2,451,288	2,452,077	+
		791 (790)	2,478,083	2,478,873	-
		789 (790)	2,481,200	2,481,988	-
ISSau4	IS3	1249 (1261)	2,416,939	2,418,187	+

Supplementary Table 5: Genomic islands in *S. epidermidis*

Island	Start	End	Integrase	No. genes	Reference
νSe1					(Gill et al., 2005)
O47	-	-	-	-	
ATCC 12228	SE0110	-	-	1	
RP62A	SERP2213	SERP2237	-	25	
νSe2/φSe1					(Gill et al., 2005; Takeuchi et al., 2005)
O47	FHQ17_05445	FHQ17_05250	FHQ17_05250	36	
ATCC 12228	SE1472	SE1509	SE1509	38	
RP62A	-	-	-	-	
νSeγ					(Gill et al., 2005)
O47	FHQ17_08705	FHQ17_08680	-	5	
ATCC 12228	SE0845	SE0850	-	5	
RP62A	SERP0735	SERP0740	-	5	
νSe3					(Takeuchi et al., 2005)
O47	FHQ17_10335	FHQ17_09985	FHQ17_10335	20	
ATCC 12228	SE0568	SE0588	SE0568	21	
RP62A	SERP0455	SERP0477	-	23	
νSe4					(Takeuchi et al., 2005)
O47	-	FHQ17_07780	-	1	

ATCC 12228	SE0988	SE0994	SE0988	7	
RP62A	SERP0878	SERP0882	-	5	
νSe5					(Takeuchi et al., 2005)
O47	FHQ17_05495	FHQ17_05445	FHQ17_05445	8	
ATCC 12228	SE1463	SE1473	SE1472	11	
RP62A	SERP1357	SERP1362	-	6	
νSe6					(Takeuchi et al., 2005)
O47	FHQ17_12035	FHQ17_12065	Fragments: FHQ17_12050- FHQ17_12060	7	
ATCC 12228	SE2339	SE2346	Fragments: SE2343 - SE2345	8	
RP62A	SERP0075	SERP0071	-	5	
νSe7					
O47	,FHQ17_12320	FHQ17_12280	-	9	
ATCC 12228	SE2395	SE2388	-	8	
RP62A	-	-	-	-	

Supplementary Table 6: *S. epidermidis* virulence factors in *S. epidermidis* O47

Gene	Locus	Best hit (strain/accession)	Product	Reference
aae	FHQ17_11920	ATCC 12228/NP_765874	bifunctional autolysin and adhesin	(Heilmann et al., 1997)
aap	FHQ17_00570	RP62A/YP_189945	accumulation associated protein	(Rohde et al., 2005)
atlE	FHQ17_09190	RP62A/YP_188221	bifunctional autolysin and adhesin	(Heilmann et al., 1997)
capB	FHQ17_01885	RP62A/YP_189663	capsule biosynthesis protein	(Kocianova et al., 2005)
capC	FHQ17_01890	ATCC 12228/NP_765647		
capA	FHQ17_01895	RP62A/YP_189661		
dltA	FHQ17_09805	ATCC 12228/NP_764179	dlt protein	(Peschel et al., 1999)
dltB	FHQ17_09800	ATCC 12228/NP_764180		
dltC	FHQ17_09795	ATCC 12228/NP_764181		
dltD	FHQ17_09790	ATCC 12228/NP_764182		
ebp	FHQ17_06935	BCM-HMP0060/ZP_04825375	elastin binding protein	(Park et al., 1996)
gehC	FHQ17_01050	RP62A/YP_189847	lipase	(Longshaw et al., 2000)
gehD	FHQ17_00610	RP62A/YP_189935		
icaA	FHQ17_01070	sp—Q8GLC5.1—ICAA_STAEP	intercellular adhesion protein	(Heilmann et al., 1996b)
icaD	FHQ17_01065	RP62A/YP_189844		
icaB	FHQ17_01060	RP62A/YP_189845		
icaC	FHQ17_01055	RP62A/YP_189846		
mprF	FHQ17_07565	RP62A/YP_188509	multiple peptide resistance factor	(Peschel et al., 2001)

oatA	FHQ17_01730	RP62A/YP_189693	O-acetyltransferase A	(Bera et al., 2006)
psmα	FHQ17_12005	RP62A/AAW55287		
psmβ1	FHQ17_08685	ATCC 12228/NP_764403		
psmβ2	FHQ17_08690	ATCC 12228/NP_764402		
psmβ3	FHQ17_08695	RP62A/YP_188319	phenol soluble modulin	(Yao et al., 2005)
hld	FHQ17_04230	ATCC 12228/NP_765189		
psmδ	FHQ17_12000			
psmε	FHQ17_10045			
sepA	FHQ17_01275	ATCC 12228/NP_765774	extracellular elastase	(Teufel and Götz, 1993)
sdrF	FHQ17_12320	ATCC 12228/NP_765950	collagen-binding protein	(Arrecubieta et al., 2007)
sdrG	FHQ17_11285	ATCC 12228/NP_763886, sp—Q9KI13.1—SDRG_STAEP	fibrinogen binding protein	(Rennermalm et al., 2004)
sdrH	FHQ17_04240	BCM-HMP0060/ZP_04824329	fibrinogen binding protein	(McCrea et al., 2000)
sitA	FHQ17_10915	W23144/ZP_04796443		
sitB	FHQ17_10920	ATCC 12228/NP_76961	iron ABC transporter protein	(Cockayne et al., 1998)
sitC	FHQ17_10925	ATCC 12228/NP_76960		
sfnaD	FHQ17_03480	RP62A/YP_189345		
sfnaA	FHQ17_03485	ATCC 12228/NP_765326		
sfnaB	FHQ17_03490	RP62A/YP_189343	staphyloferrin A biosynthesis protein	(Lindsay et al., 1994; Cotton et al., 2009)
sfnaC	FHQ17_03495	ATCC 12228/NP_765324		
sspA	FHQ17_05050	ATCC 12228/NP_765098	serine protease	

sspB	FHQ17_00605	ATCC 12228/NP_763739	cysteine protease	(Dubin et al., 2001)
tarI	FHQ17_11345	ATCC 12228/NP_763874	teichoic acid biosynthesis protein	(Fitzgerald and Foster, 2000; Weidenmaier et al., 2004; Qian et al., 2006; Brown et al., 2008; Swoboda et al., 2010; Holland et al., 2011)
tarJ	FHQ17_11340	ATCC 12228/NP_763875		
tarL	FHQ17_11335	BCM-HMP0060/ZP_04824448		
tagA	FHQ17_10900	ATCC 12228/NP_763965		
tagH	FHQ17_10895	BCM-HMP0060/ZP_04824541		
tagG	FHQ17_10890	ATCC 12228/NP_763867		
tagB	FHQ17_10885	ATCC 12228/NP_763868		
tagX	FHQ17_10880	ATCC 12228/NP_763869		
tagD	FHQ17_10875	ATCC 12228/NP_763870		
tagO	FHQ17_10310	BCM-HMP0060/ZP_04824654		
tagF	FHQ17_02600	M23864:W2(grey)/ZP_06613749		
tagE	FHQ17_02150	ATCC 12228/NP_765596		
vraG	FHQ17_10795	ATCC 12228/NP_763984	ABC transporter	(Li et al., 2007)
vraF	FHQ17_10800	ATCC 12228/NP_763985		

Supplementary Table 7: Penicillin binding proteins in *S. epidermidis*

**Gene**	**Product**	**Locus**		
		**O47**	**RP62A**	**ATCC 12228**
pbp1	penicillin binding protein 1	FHQ17_08650	SERP0746	SE0856
pbp2	penicillin binding protein 2	FHQ17_07075	SERP1020	SE1138
pbp3	penicillin binding protein 3	FHQ17_06590	SERP1117	SE1238
pbp4	penicillin binding protein 4	-	-	SE0035

Supplementary Table 8: MIC values* for *S. epidermidis* O47, ATCC 12228 and RP62A and *S. carnosus* TM300 and *S. aureus* USA300.

**Strains**	**MIC (µg/ml)**		
	Penicillin	Methicillin	Fosfomycin
Staphylococcus carnosus TM300	Sensitive (< 0.5)	4	4
Staphylococcus epidermidis O47	>128	2	8
Staphylococcus epidermidis 12228	>128	4	2
Staphylococcus epidermidis RP62A	>128	>128	1
Staphylococcus aureus USA300	64	>128	8

* MIC determinations were performed in three independent biological replicates.

Supplementary Table 9: Resistances in *S. epidermidis* O47

Gene	Locus	Product
	FHQ17_12065	COG: Abortive infection bacteriophage resistance protein
uppP	FHQ17_10675	COG: Uncharacterized bacitracin resistance protein
norA	FHQ17_10615	quinolone resistance protein
	FHQ17_10050	cobalt-zinc-cadmium resistance protein
	FHQ17_09350	COG: Membrane protein TerC, possibly involved in tellurium resistance
	FHQ17_08020	COG: Cystathionine beta-lyase family protein involved in aluminum resistance
	FHQ17_07320	COG: Uncharacterized protein involved in tellurite resistance
cadD	FHQ17_05180	cadmium resistance transporter CadD
vraR	FHQ17_04555	K07694 two-component system, NarL family,vancomycin resistance associated response regulator VraR
vraS	FHQ17_04550	K07681 two-component system, NarL family,vancomycin resistance sensor histidine kinase VraS
	FHQ17_03555	EmrB/QacA family drug resistance transporter
fmhB	FHQ17_03175	COG: Uncharacterized protein involved in methicillin resistance
ykkC	FHQ17_02955	K11741 quaternary ammonium compound-resistance protein SugE
	FHQ17_02950	K11741 quaternary ammonium compound-resistance protein SugE
	FHQ17_02675	COG: Multidrug resistance efflux pump
tcaB	FHQ17_02665	
tcaA	FHQ17_02660	teicoplanin-associated operon
tcaR	FHQ17_02655	
	FHQ17_02555	COG: Uncharacterized protein, homolog of Cu resistance protein CopC
fmhA	HQ17_02405	COG: Uncharacterized protein involved in methicillin resistance
bcr	FHQ17_02350	bicyclomycin resistance protein

	FHQ17_02310	K08170 MFS transporter, DHA2 family, multidrug resistance protein
ohr	FHQ17_01205	organic hydroperoxide resistance protein
	FHQ17_00650	K08170 MFS transporter, DHA2 family, multidrug resistance protein
arsR	FHQ17_00425	
arsD	FHQ17_00415	
arsA	FHQ17_00415	arsenical resistance operon
arsR	FHQ17_00410	
arsB	FHQ17_00405	
arsC	FHQ17_00400	
merB	FHQ17_00350	
merA	FHQ17_00345	mercuric resistance operon
merR	FHQ17_00325	
azlC	FHQ17_00055	azaleucine resistance
azlD	FHQ17_00060	
